# Supplementary material for: Rare Earth Elements in Boletus edulis (King Bolete) Mushrooms from Lowland and Montane Areas in Poland
Source: Int J Environ Res Public Health. 2022 Jul 22;19(15):8948. doi: 10.3390/ijerph19158948 (PMC9331855; doi:10.3390/ijerph19158948)
Supplement: Supplementary file 1 [file ijerph-19-08948-s001.zip › ijerph-1769630-supplementary.pdf]

Supplementary Material

Rare earth elements in Boletus edulis (king bolete) from lowland and montane areas

Jerzy Falandysz[1*], Nnorom Innocent Chidi[2], Małgorzata Mędyk[1]

[1]Medical University of Łódź, Faculty of Pharmacy, Department of Toxicology,

1 Muszyńskiego Street, 90-151 Łódź, Poland

[2]Abia State University, Department of Pure and Industrial Chemistry, Analytical / Environmental Unit, Uturu, Nigeria

[*]Corresponding author e-mail: jerzy.falandysz@umed.lodz.pl

Jerzy Falandysz: ORCID 0000-0003-2547-2496

Nnorom Innocent Chidi: ORCID 0000-0001-8801-8931

Table S1. The NASC-, PAAS-, EUS- and WSH-normalized REE concentrations in the caps, stipes and whole fruiting bodies of *B. edulis* (whole set)

REE	REE concentrations (mg kg^{-1})				Caps: Normalization				Stipes: Normalization				Whole: Normalization			
	NASC[a]	PAAS[b]	EUS[c]	WSH[d]	NASC	PAAS	EUS	WSH	NASC	PAAS	EUS	WSH	NASC	PAAS	EUS	WSH
La	31.1	44.54	44.3	40.3	0.00106	0.00074	0.00074	0.00082	0.00180	0.00126	0.00126	0.00139	0.00164	0.00145	0.00115	0.00126
Ce	67.033	88.25	88.5	83.3	0.00089	0.00068	0.00068	0.00072	0.00164	0.00125	0.00124	0.00132	0.00142	0.00108	0.00107	0.00114
Pr	7.9	10.15	10.6	9.54	0.00081	0.00063	0.00060	0.00067	0.00139	0.00108	0.00104	0.00115	0.00123	0.00096	0.00091	0.00102
Nd	30.4	37.32	39.5	31.6	0.00072	0.00059	0.00056	0.00070	0.00135	0.00110	0.00104	0.00130	0.00105	0.00086	0.00081	0.00101
Sm	5.98	6.884	7.30	7.30	0.00057	0.00049	0.00047	0.00047	0.00105	0.00091	0.00086	0.00086	0.00092	0.00080	0.00075	0.00075
Eu	1.253333	1.215	1.48	1.31	0.00048	0.00049	0.00040	0.00046	0.00072	0.00074	0.00061	0.00068	0.00088	0.00090	0.00074	0.00084
Gd	5.5	6.043	6.34	5.86	0.00078	0.00071	0.00068	0.00073	0.00094	0.00086	0.00082	0.00089	0.00093	0.00084	0.00080	0.00087
Tb	0.85	0.891	0.944	0.90	0.00082	0.00079	0.00074	0.00078	0.00141	0.00135	0.00127	0.00133	0.00110	0.00101	0.00095	0.00100
Dy	5.75	5.325	5.86	5.66	0.00066	0.00071	0.00065	0.00067	0.00096	0.00103	0.00094	0.00097	0.00087	0.00094	0.00085	0.00088
Y	27	27.31	31.9	28.7	0.00115	0.00113	0.00097	0.00108	0.00155	0.00157	0.00135	0.0015	0.00155	0.00154	0.00132	0.00146
Ho	1.2	1.053	1.17	1.09	0.00058	0.00066	0.00060	0.00064	0.00092	0.00104	0.00094	0.00101	0.00083	0.00095	0.00085	0.00092
Er	3.275	3.075	3.43	3.30	0.00064	0.00068	0.00061	0.00064	0.00110	0.00117	0.00105	0.00109	0.00104	0.00111	0.00099	0.00103
Tm	0.5	0.451	0.492	0.48	0.00080	0.00089	0.00081	0.00083	0.00100	0.00111	0.00102	0.00104	0.00120	0.00133	0.00122	0.00125
Yb	3.113333	3.012	3.26	3.12	0.00064	0.00066	0.00061	0.00064	0.00112	0.00116	0.00107	0.00112	0.00103	0.00106	0.00098	0.00103
Lu	0.456	0.4386	0.485	0.47	0.00088	0.00114	0.00082	0.00085	0.00110	0.00114	0.00103	0.00106	0.00110	0.00114	0.00103	0.00106

Notes: a (North American Shale Composite), b (Post-Archean Australian Shales), c (European Shale), d (World Shale).

Table S2. NASC-, PAAS-, EUS- and WSH-normalized REE concentrations in the whole fruiting bodies of *B. edulis* from the Morąg and Augustów sites in this study and a single specimen of *B. edulis* examined by Bau et al. (2018)

REE	REE concentrations (mg kg^{-1})				Whole (Morąg; id8): Normalization				Whole (Augustów; id6): Normalization				Whole (Bau et al. 2018) Normalization			
	NASC[a]	PAAS[b]	EUS[c]	WSH[d]	NASC	PAAS	EUS	WSH	NASC	PAAS	EUS	WSH	NASC	PAAS	EUS	WSH
La	31.1	44.56	44.3	40.3	0.00965	0.00673	0.00677	0.00744	0.00482	0.00337	0.00339	0.00372	0.00047	0.00032	0.00033	0.00036
Ce	67.033	88.25	88.5	83.3	0.00940	0.00714	0.00712	0.00756	0.00403	0.00306	0.00305	0.00324	0.00036	0.00027	0.00027	0.00029
Pr	7.9	10.15	10.6	9.54	0.00797	0.00621	0.00641	0.00660	0.00329	0.00256	0.00245	0.00272	0.00036	0.00028	0.00026	0.00029
Nd	30.4	37.32	39.5	31.6	0.00789	0.00643	0.00608	0.00759	0.00260	0.00212	0.00200	0.00250	0.00033	0.00027	0.00026	0.00032
Sm	5.98	6.884	7.30	7.30	0.00736	0.00639	0.00603	0.00603	0.00167	0.00145	0.00137	0.00137	0.00038	0.00033	0.00031	0.00031
Eu	1.253333	1.215	1.48	1.31	0.00774	0.00798	0.00655	0.00740	0.00271	0.00280	0.00230	0.00260	0.00042	0.00042	0.00035	0.00040
Gd	5.5	6.043	6.34	5.86	0.00654	0.00596	0.00678	0.00614	0.00153	0.00139	0.00132	0.00143	0.00050	0.00045	0.00043	0.00047
Tb	0.85	0.891	0.944	0.90	0.00729	0.00696	0.00657	0.00689	0.00164	0.00157	0.00148	0.00155	0.00045	0.00043	0.00041	0.00043
Dy	5.75	5.325	5.86	5.66	0.00574	0.00620	0.00563	0.00583	0.00125	0.00135	0.00123	0.00127	0.00040	0.00043	0.00039	0.00041
Y	27	27.32	31.9	28.7	0.01148	0.01135	0.00972	0.0108	0.00259	0.00256	0.00219	0.00244	0.00067	0.00066	0.00056	0.00063
Ho	1.2	1.053	1.17	1.09	0.00608	0.00693	0.00624	0.00670	0.00133	0.00152	0.00137	0.00147	0.00036	0.00041	0.00037	0.00040
Er	3.275	3.075	3.43	3.30	0.00641	0.00683	0.00612	0.00636	0.00165	0.00176	0.00157	0.00164	0.00036	0.00038	0.00034	0.00036
Tm	0.5	0.451	0.492	0.48	0.00700	0.00776	0.00711	0.00729	0.00120	0.00133	0.00122	0.00125	WD	WD	WD	WD
Yb	3.113333	3.012	3.26	3.12	0.00771	0.00797	0.00736	0.00769	0.00138	0.00143	0.00132	0.00138	0.00029	0.00030	0.00028	0.00029
Lu	0.456	0.4386	0.485	0.47	0.00789	0.00821	0.00742	0.00766	0.00153	0.00160	0.00144	0.00149	0.00026	0.00028	0.00025	0.00026

Notes: a (North American Shale Composite), b (Post-Archean Australian Shales), c (European Shale), d (World Shale), WD (without data).

FIGURES

REY

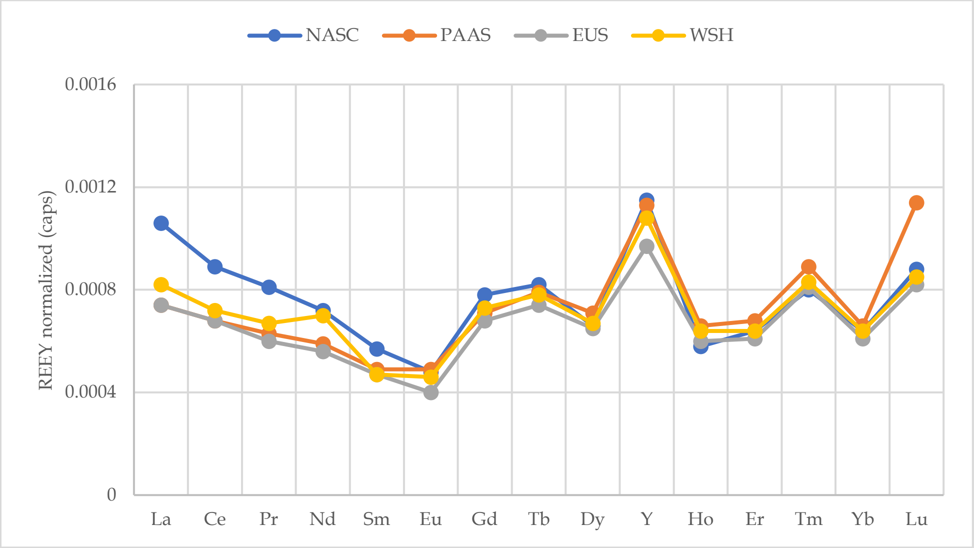

(a; caps)

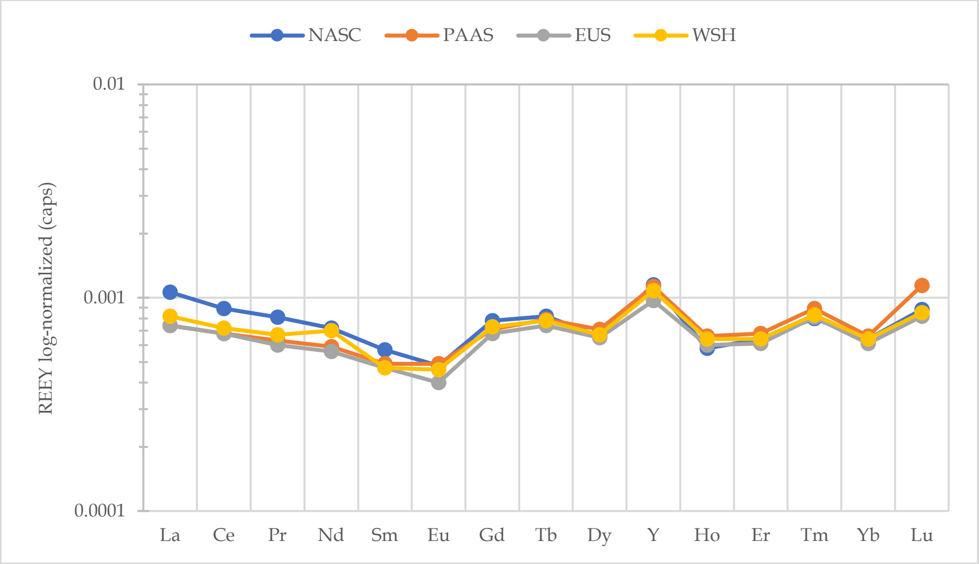

(a; caps)

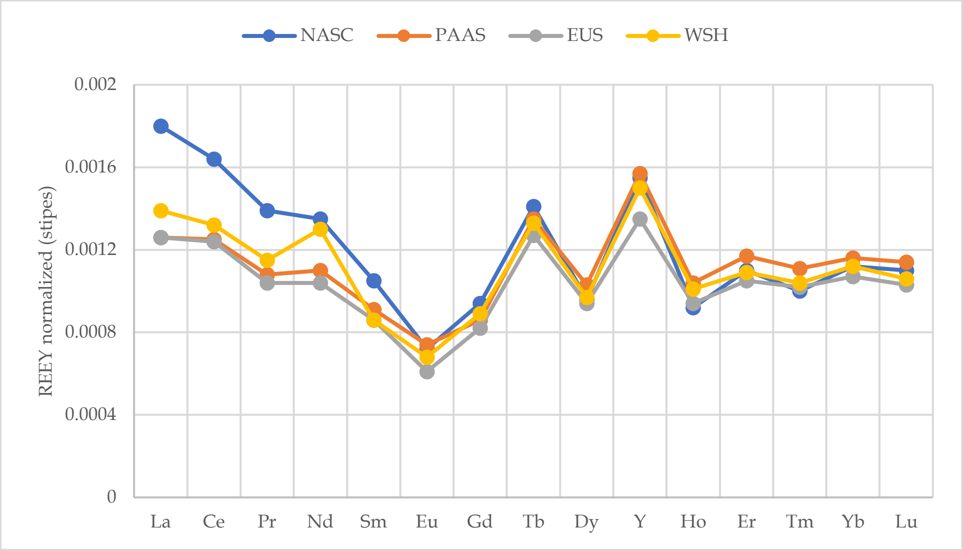

(b; stipes)

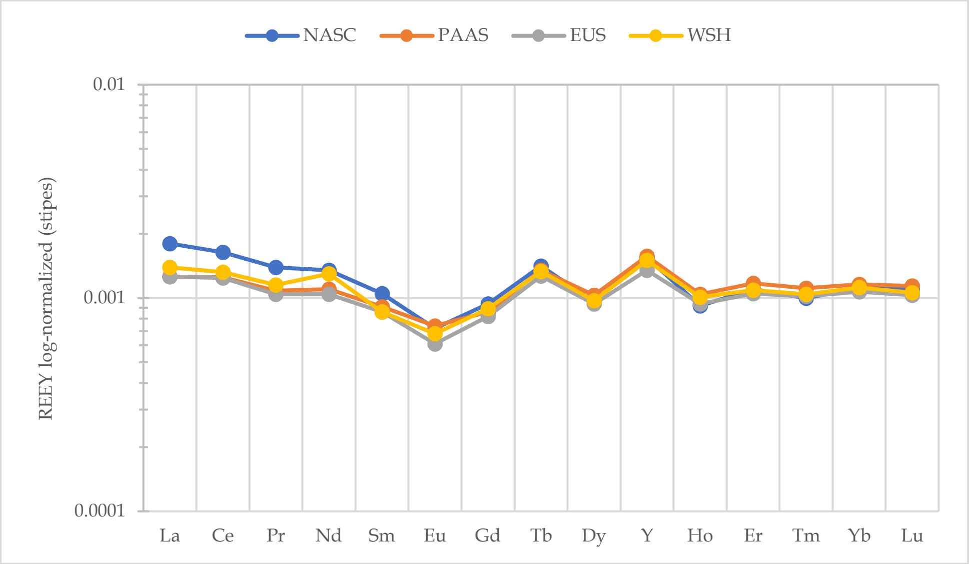

(b; stipes)

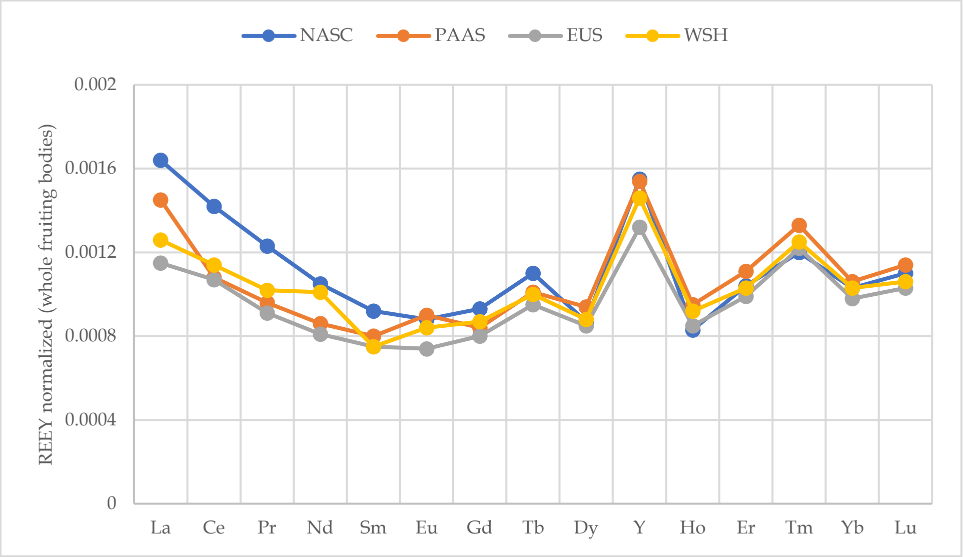

(c; whole)

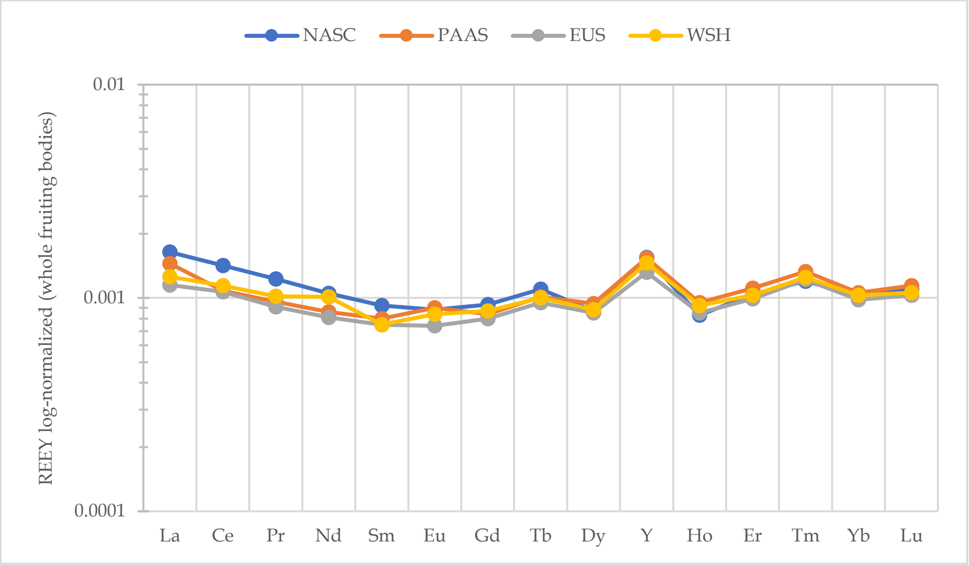

(c; whole)

Figure 1. The shale (NASC, PAAS, EUS and WSH) normalised patterns of REY in *B. edulis* [medians for the caps (a), stipes (b) and the whole fruiting bodies (c)].

REE

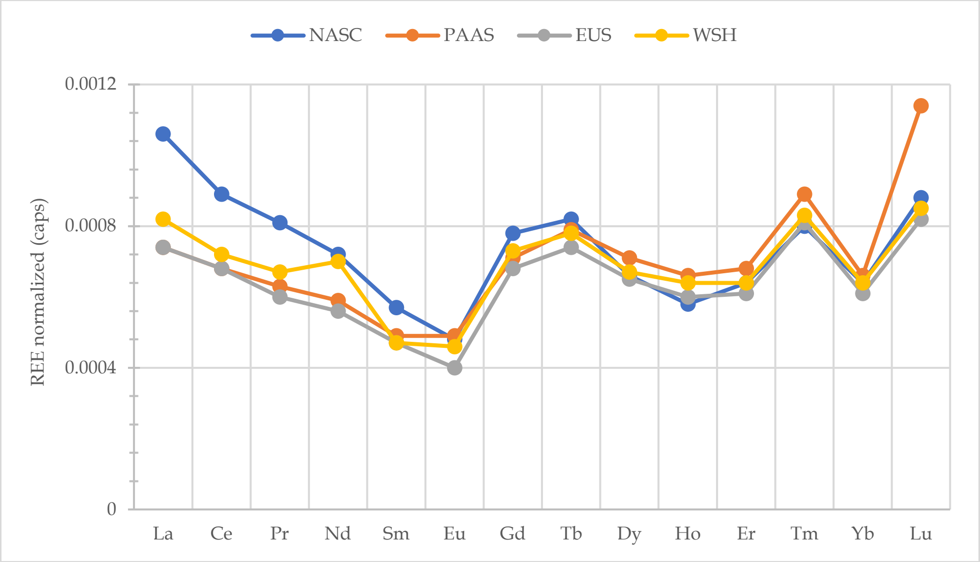

(a; caps)

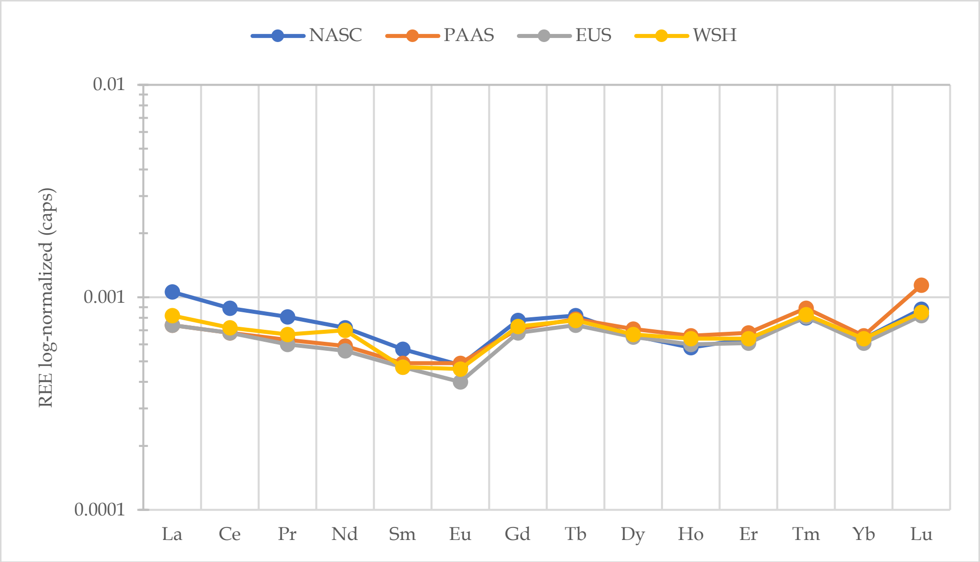

(a; caps)

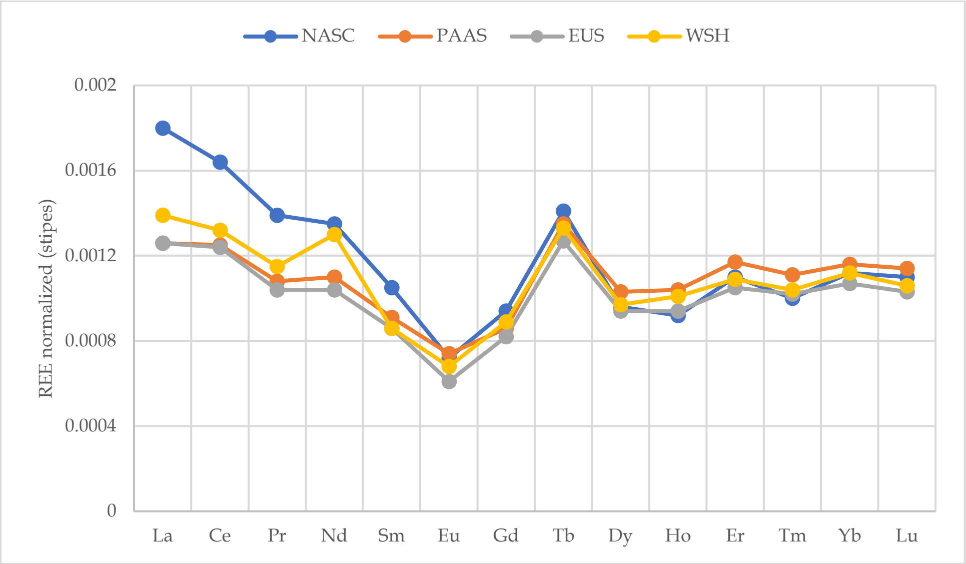

(b; stipes)

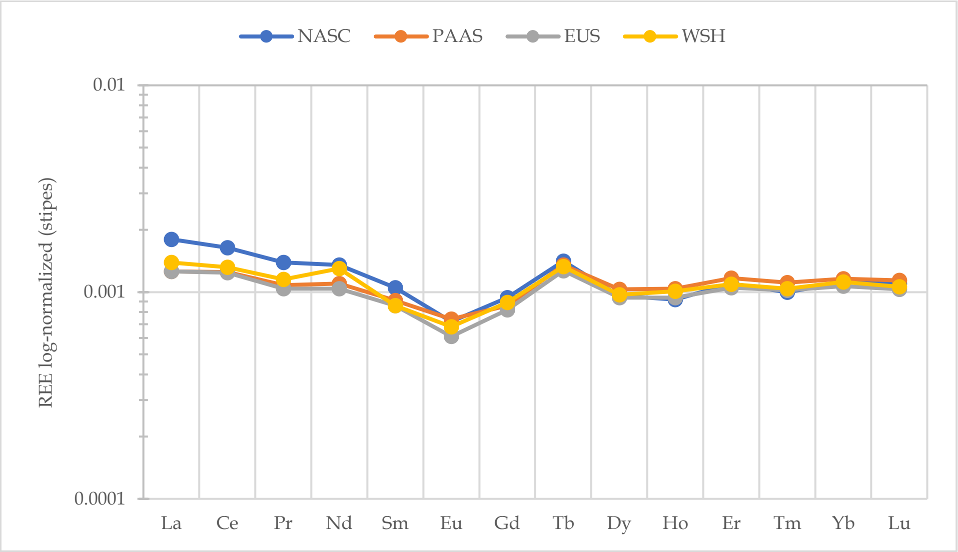

(b; stipes)

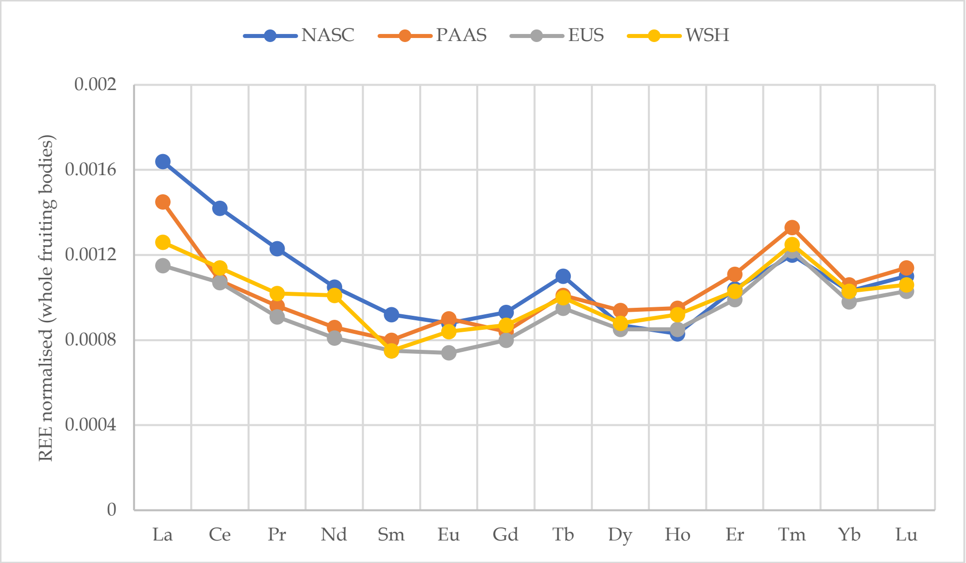

(c; whole)

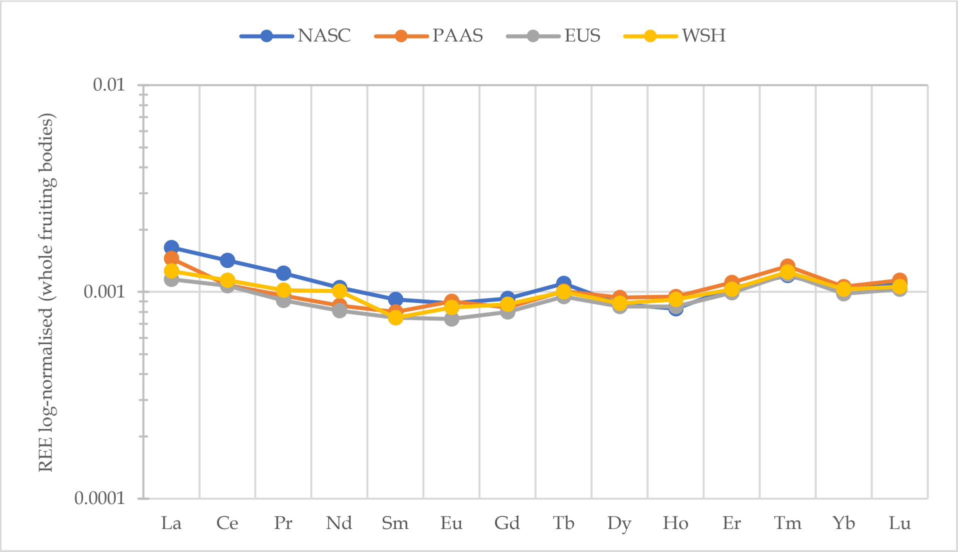

(c; whole)

Figure 2. The shale (NASC, PAAS, EUS and WSH) normalised patterns of REE in *B. edulis* [medians for the caps (a), stipes (b) and the whole fruiting bodies (c)].

REY

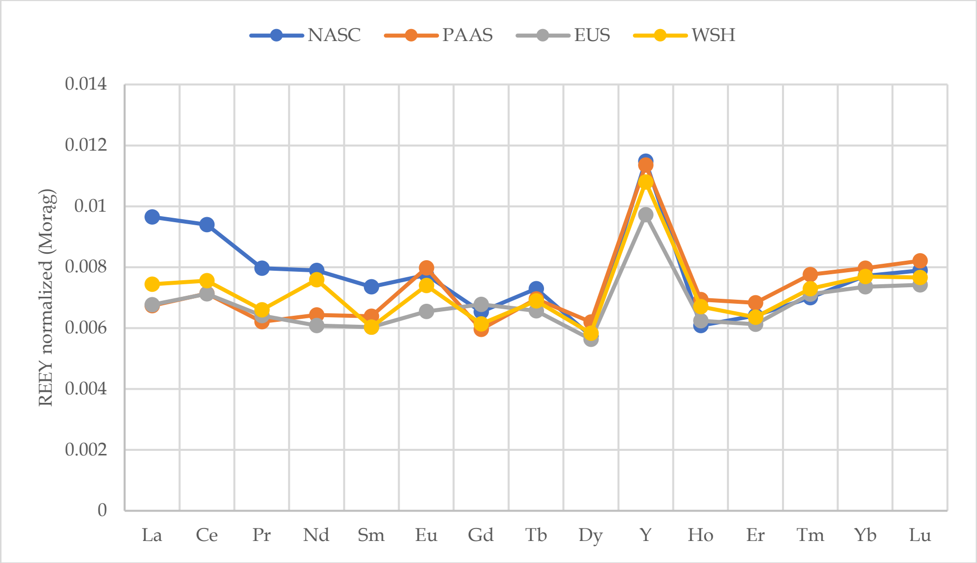

(a; Morąg site, whole fruiting bodies)

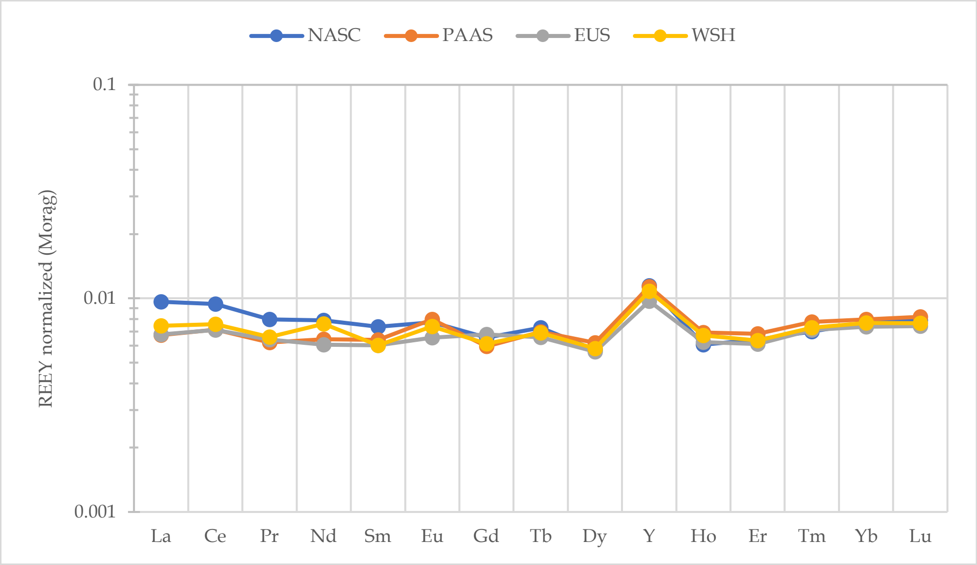

(a; Morąg site, whole fruiting bodies)

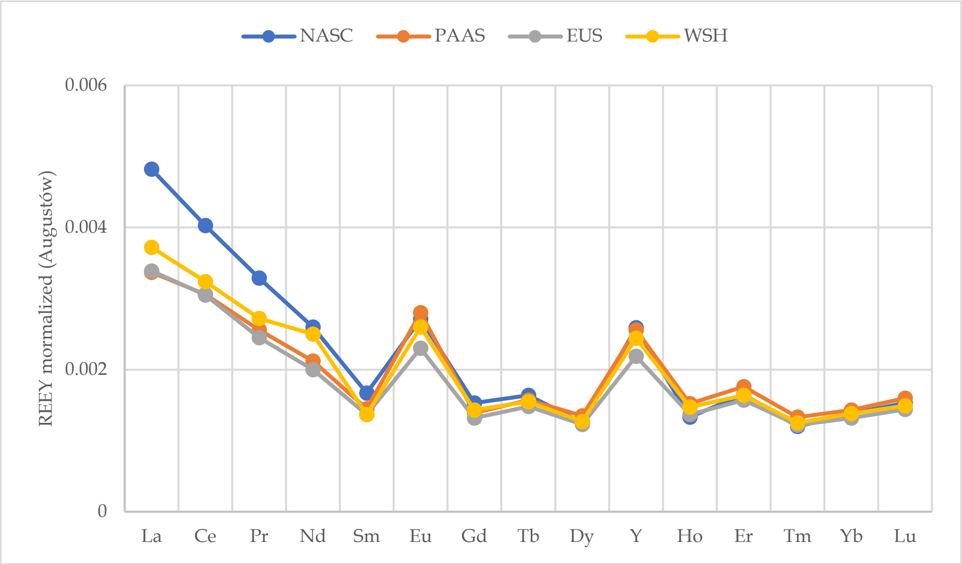

(b; Augustów Primeval Forest, whole fruiting bodies)

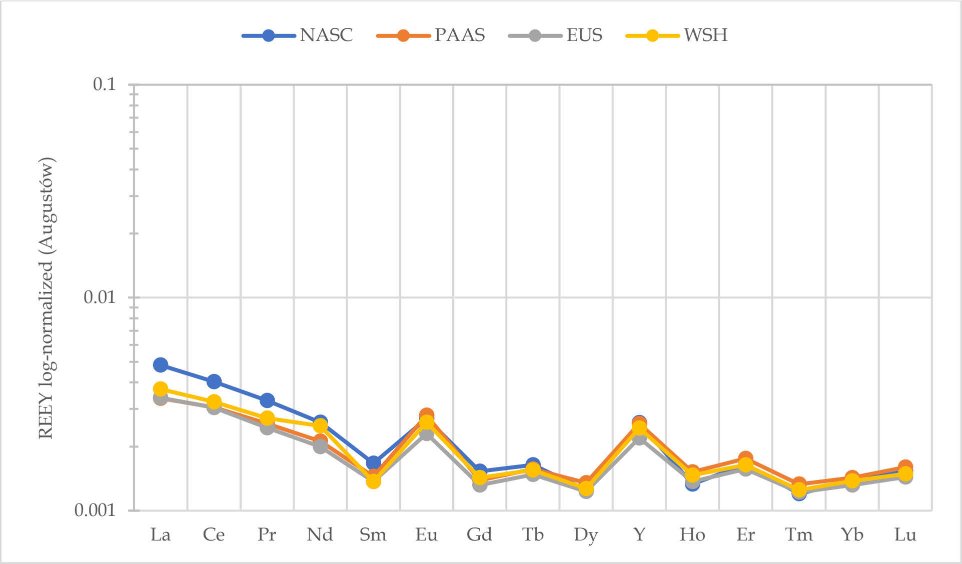

(b; Augustów Primeval Forest, whole fruiting bodies)

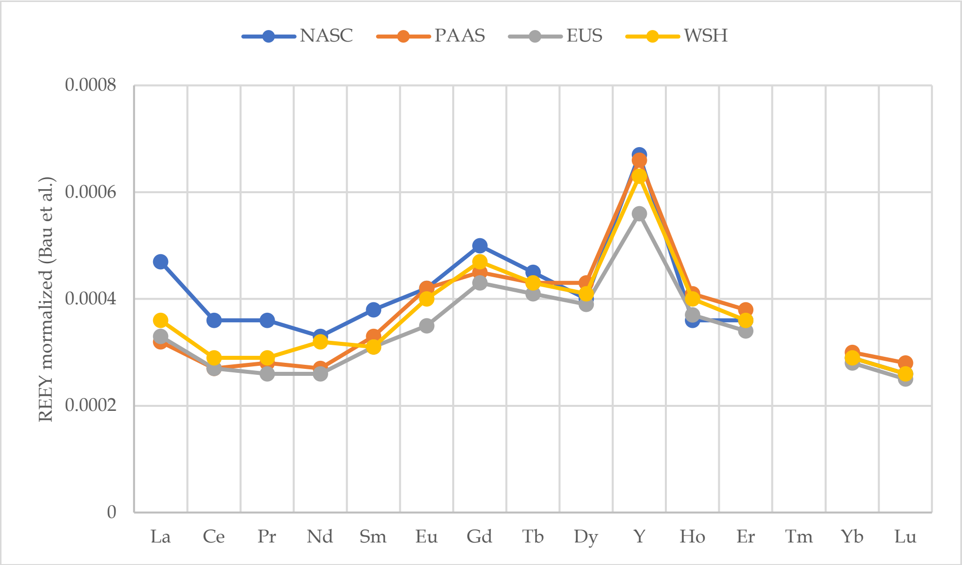

(c) a single *B. edulis* carpophore (Bau et al., 2018)

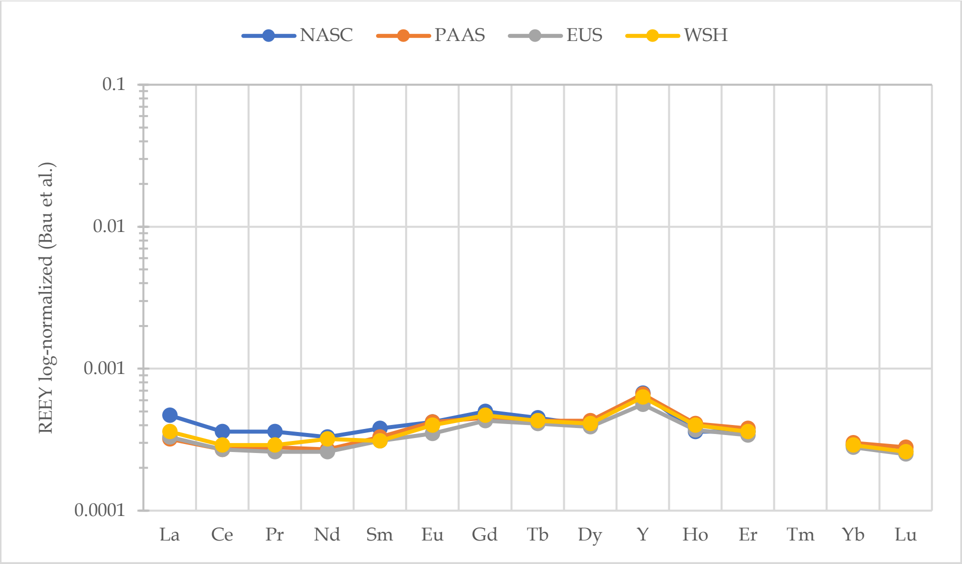

(c) a single *B. edulis* carpophore (Bau et al., 2018)

Figure 3. Shale (NASC, PAAS, EUS and WSH) log-normalised patterns of REY in *B. edulis* (whole fruiting bodies) from the sites (a) Morąg and (b) Augustów, and (c) Bau et al. (2018).

REE

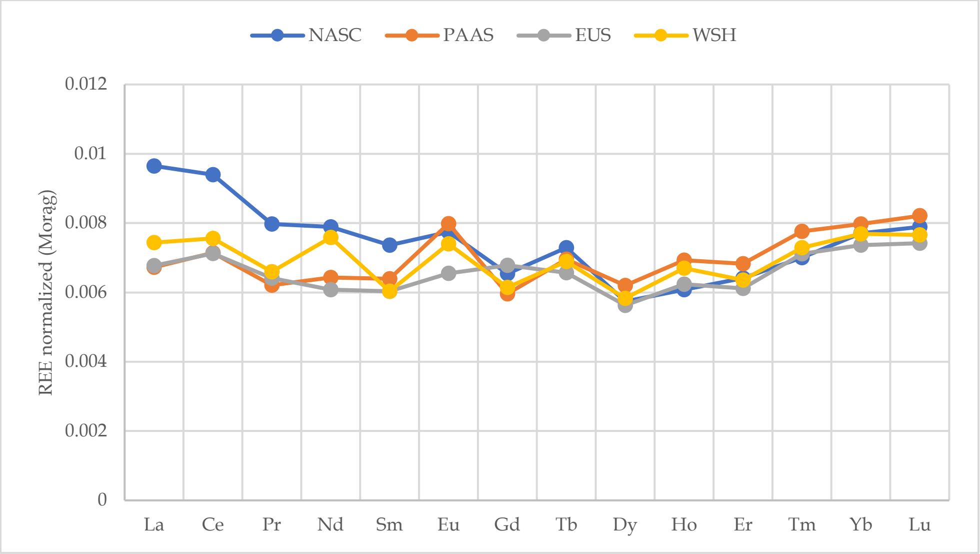

(a; Morąg site, whole fruiting bodies)

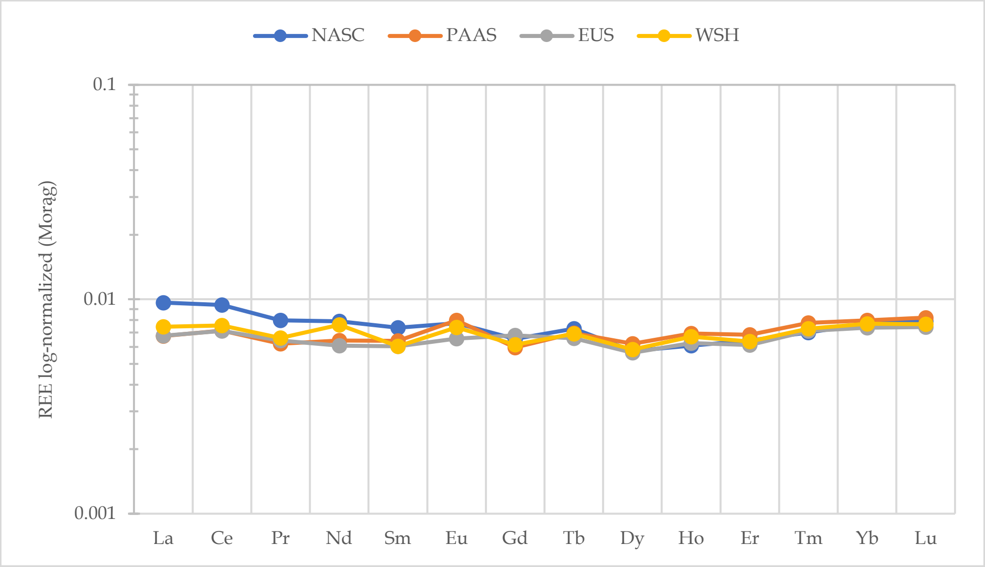

(a; Morąg site, whole fruiting bodies)

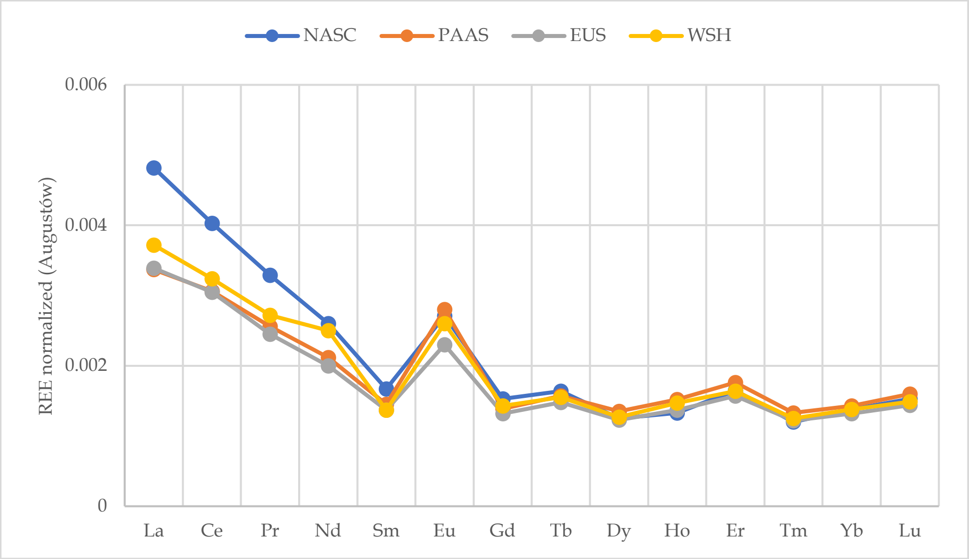

(b; Augustów Primeval Forest, whole fruiting bodies)

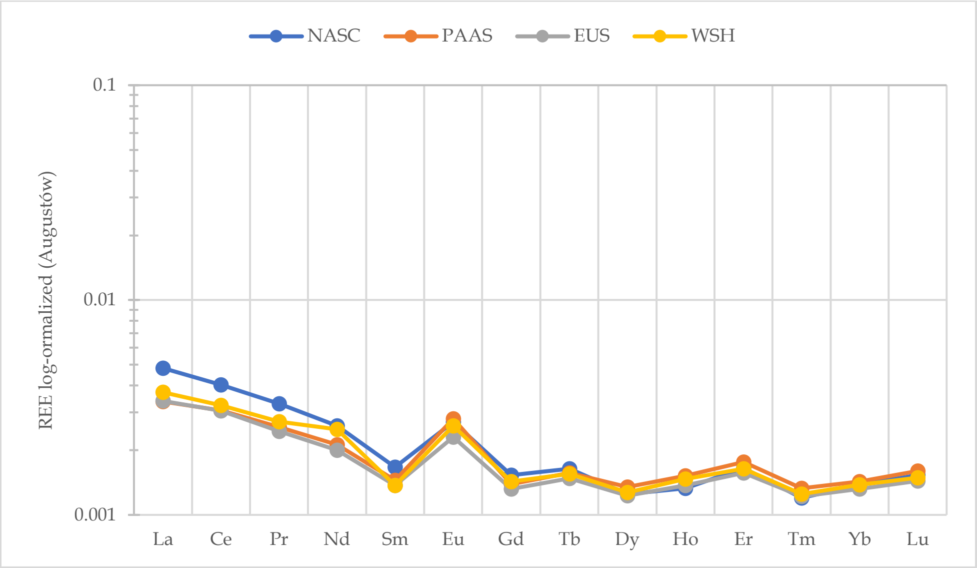

(b; Augustów Primeval Forest, whole fruiting bodies)

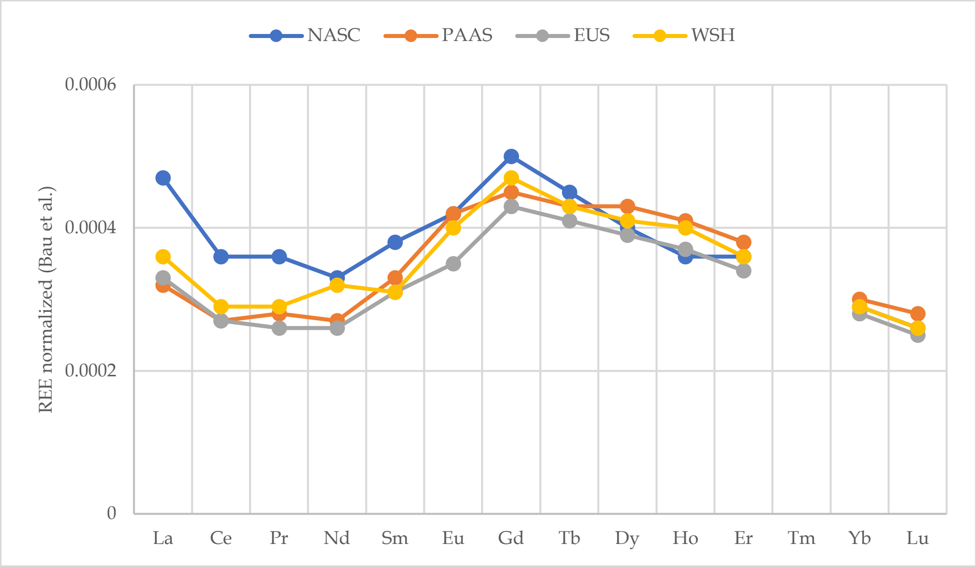

(c) a single *B. edulis* carpophore (Bau et al., 2018)

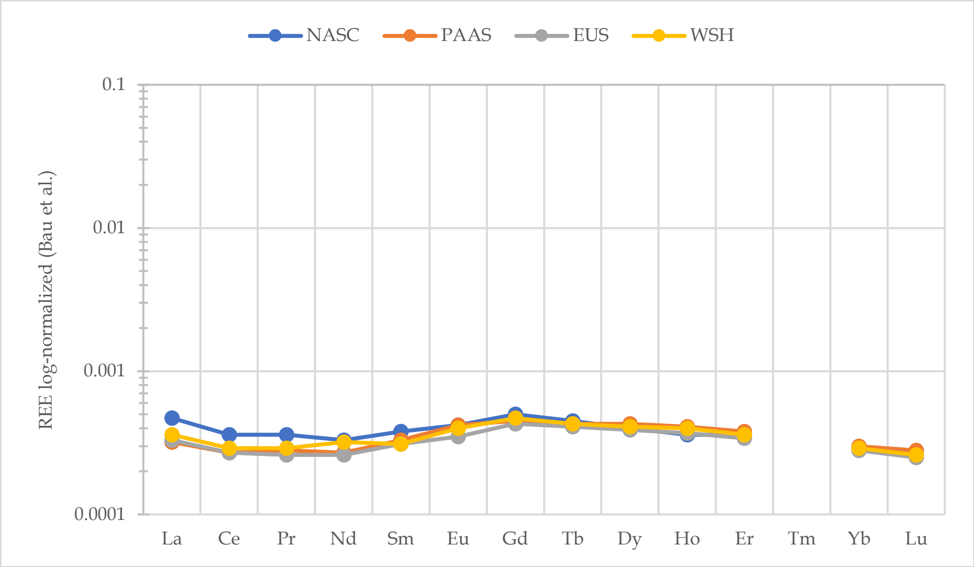

(c) a single *B. edulis* carpophore (Bau et al., 2018)

Figure 4. Shale (NASC, PAAS, EUS and WSH) log-normalised patterns of REE in *B. edulis* (whole fruiting bodies) from the sites (a) Morąg and (b) Augustów, and (c) Bau et al. (2018).
